# Urinary proteomics can define distinct diagnostic inflammatory arthritis subgroups

**DOI:** 10.1038/srep40473

**Published:** 2017-01-16

**Authors:** Stefan Siebert, Duncan Porter, Caron Paterson, Rosie Hampson, Daniel Gaya, Agnieszka Latosinska, Harald Mischak, Joost Schanstra, William Mullen, Iain McInnes

**Affiliations:** 1 Institute of Infection, Immunity and Inflammation, College of Medical, Veterinary and Life Sciences, University of Glasgow, Glasgow, United Kingdom; 2 Rheumatology Department, NHS Greater Glasgow and Clyde, Glasgow, UK; 3 Gastroenterology Department, NHS Greater Glasgow and Clyde, Glasgow, UK; 4 Mosaiques Diagnostics, Hannover, Germany; 5 Institute of Cardiovascular and Medical Sciences, University of Glasgow, Glasgow, UK; 6 Institut National de la Santé et de la Recherche Médicale (INSERM), U1048, Institute of Cardiovascular and Metabolic Disease, Toulouse, France; 7 Université Toulouse III Paul-Sabatier, Toulouse, France

## Abstract

Current diagnostic tests applied to inflammatory arthritis lack the necessary specificity to appropriately categorise patients. There is a need for novel approaches to classify patients with these conditions. Herein we explored whether urinary proteomic biomarkers specific for different forms of arthritis (rheumatoid arthritis (RA), psoriatic arthritis (PsA), osteoarthritis (OA)) or chronic inflammatory conditions (inflammatory bowel disease (IBD)) can be identified. Fifty subjects per group with RA, PsA, OA or IBD and 50 healthy controls were included in the study. Two-thirds of these populations were randomly selected to serve as a training set, while the remaining one-third was reserved for validation. Sequential comparison of one group to the other four enabled identification of multiple urinary peptides significantly associated with discrete pathological conditions. Classifiers for the five groups were developed and subsequently tested blind in the validation test set. Upon unblinding, the classifiers demonstrated excellent performance, with an area under the curve between 0.90 and 0.97 per group. Identification of the peptide markers pointed to dysregulation of collagen synthesis and inflammation, but also novel inflammatory markers. We conclude that urinary peptide signatures can reliably differentiate between chronic arthropathies and inflammatory conditions with discrete pathogenesis.

Early diagnosis of inflammatory arthritis offers a window of opportunity for effective treatment and is associated with improved patient outcomes^1^,^2^. However, in the initial stages of the disease, accurate diagnosis can be challenging because of the lack of sufficiently sensitive and specific diagnostic tests. Rheumatoid factor and anti-citrullinated protein antibodies used to support the diagnosis of rheumatoid arthritis (RA) have limited selectivity^3^. Moreover, there are no specific diagnostic tests available for conditions like psoriatic arthritis (PsA). Similarly, inflammatory markers such as C-reactive protein (CRP) and erythrocyte sedimentation rate (ESR) can be affected by a large number of variables, including age and obesity, and as such offer limited specificity in patient identification. Therefore, there is a need for novel diagnostic biomarkers to assist accurate, early diagnosis, particularly in primary care settings where specialist expertise and imaging may not be readily available.

We previously described the application of capillary electrophoresis coupled to mass spectrometry to identify urinary biomarkers for the diagnosis of several diseases such as acute and chronic kidney disease^4^, left ventricular dysfunction^5^, preeclampsia^6^ and cardiovascular diseases^7^, allowing accurate classification of case versus control groups. The use of urine for the identification of biomarkers has several advantages, including the non-invasive method of sample collection, the low dynamic range of analytes which facilitates the detection and analysis of biomarkers, and high stability due to absence of proteolytic agents^8^,^9^. Stability of urine and interference of different agents has been investigated recently^10^, demonstrating good reproducibility and stability. The latter allows a urine sample to be posted to a laboratory for testing rather than a patient being required to attend a primary care location for diagnosis by a specialist. Although concentration of compounds in urine differs substantially depending on the fluid intake, this can be compensated based on a set of urinary “housekeeping” peptides^11^. Furthermore, identification of proteomic biomarkers may be useful in understanding the molecular mechanisms involved in the onset and progression of a disease^12^. Disadvantages of urine may be a bias towards diseases of the kidney and urogenital tract (proteins and peptides derived thereof represent about 70% of the urine proteome), and frequently a lack of a direct connection between the biomarkers identified in urine, and the molecular changes in the tissue (not applicable for kidney disease).

In a pilot study we defined peptide biomarkers in urine that distinguished RA from healthy controls^13^. It is possible that the biomarkers identified reflected the presence of systemic inflammation, synovial inflammation, joint damage, or a combination of these. As such, they could be markers of articular insult/damage in general (conferring no diagnostic specificity), inflammatory arthritis, systemic inflammation or they could be disease specific.

In this further pilot study, we sought to identify novel urinary biomarkers that distinguish different forms of chronic arthropathy including RA, PsA, osteoarthritis (OA) from a discrete inflammatory disease, namely inflammatory bowel disease (IBD). We hypothesized that specific peptides could be detected in urine samples of patients that could be combined into a classifier, which may be developed into a tool enabling early and differential diagnosis. In order to eliminate non-specific biomarkers of classical systemic inflammation and/or joint damage, we selected diagnostic groups that over-lapped for each of these domains – i.e. OA, PsA and RA are associated with joint damage (but IBD is not); RA, PsA and IBD are chronic inflammatory diseases (but OA is not); and PsA usually involves the skin whereas RA, IBD and OA usually do not.

## Results

**Figure 1:**
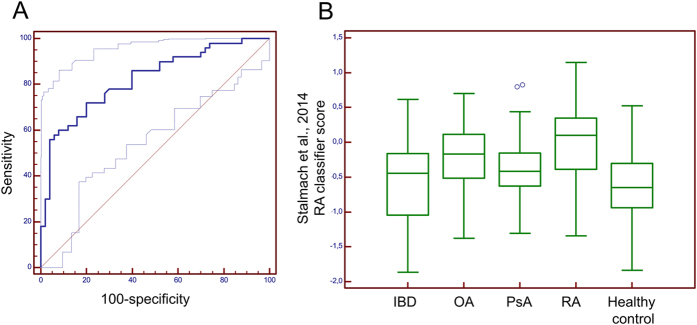
(**A**) Receiver operating characteristics (ROC) curve for the performance of the previously published biomarker model^13^ in patients with RA compared to healthy controls. The dotted lines represent the upper and lower limits of the 95% CI. (**B**) Box-whisker blots of the scoring of the 5 different groups with the previously developed “RA” classifier. IBD – inflammatory bowel disease; OA – osteoarthritis; PsA – psoriatic arthritis; RA – rheumatoid arthritis.

The demographic and applicable clinical characteristics of the cohort are shown in Table 1. All urine samples could be successfully analyzed and passed quality control^10^. As a first step we aimed at assessing the performance of the previously identified biomarker panel for RA^13^ in this independent cohort. For this purpose, the previously defined classifier was applied blinded to 100 samples from patients with RA (n = 50) and healthy controls (n = 50). Upon unblinding, the data revealed a significant association of the previously developed classifier with RA in comparison to healthy controls, with an area under the curve (AUC) of 0.83 (p < 0.01, Fig. 1A). While this confirmed the validity of the previously developed classifier for RA, it also indicated that further improvement would be beneficial for future clinical implementation.

**Figure 2:**
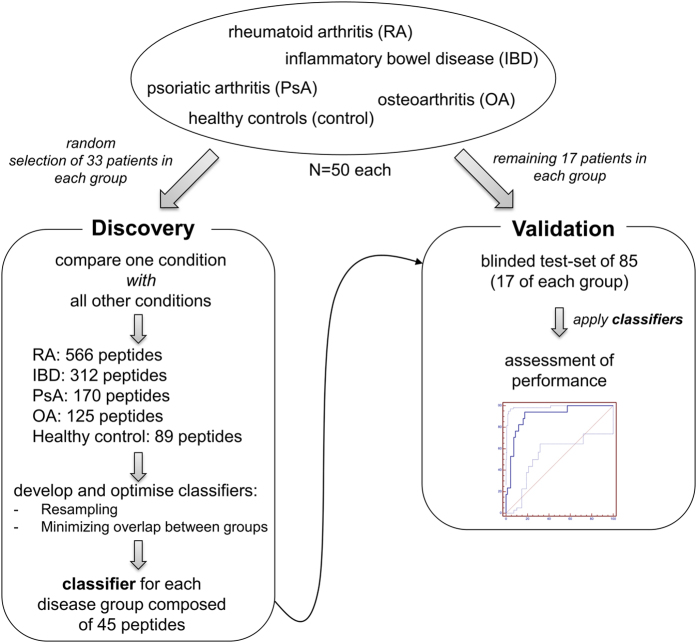
Schematic of the study design. The available samples from each of the 5 groups were randomly assigned, using an automated algorithm, to the training (discovery) or the validation test set. Samples in the training set were used to identify potential biomarkers by comparing each group with all the others to identify peptides significantly associated with each group. In order to avoid introducing bias and to give similar weighting to each group, the 50 most significant peptides in each group were then used to generate a 45 peptide classifier specific for the respective group. Performance of this classifier was tested in the remaining blinded independent samples in the validation set to give an assessment of the performance of the classifiers for each disease (shown in Fig. 4).

The previously described biomarker classifier was not developed for differential diagnosis of RA, OA, IBD, and PsA. This is also evident from the scoring using the previously developed RA classifier for these groups of patients. As shown in Fig. 1B, the scoring for the other conditions was distributed between healthy controls and RA. Differential diagnosis would require employing different classifiers, one for each disease. As a next step, we therefore investigated if the different diseases represented here can be distinguished from each other, based on the urine proteome by a *de novo* analysis. To identify biomarkers that may enable differential diagnosis, and to assess the value of such biomarkers and any classifiers developed, we employed a study design based on an initial discovery cohort employed as training set for the classifier, and another test cohort to assess the performance of the biomarkers. As depicted in Fig. 2, the cohort was separated into a training set which included a random selection of 2 thirds of the entire cohort (33 samples from each of the four disease and the healthy control groups), and a validation test set, containing 1/3 of the samples (n = 17 per group).

As a first step we aimed at identifying urinary biomarkers specific for the 5 different clinical groups. To this end, we compared each group (PsA, RA, OA, IBD, healthy controls) with all others. In each case we could identify peptides significantly associated with the respective group being tested, ranging between 89 (healthy controls) and 566 (RA) peptides. The results are presented in Supplementary Table 1.

**Figure 3:**
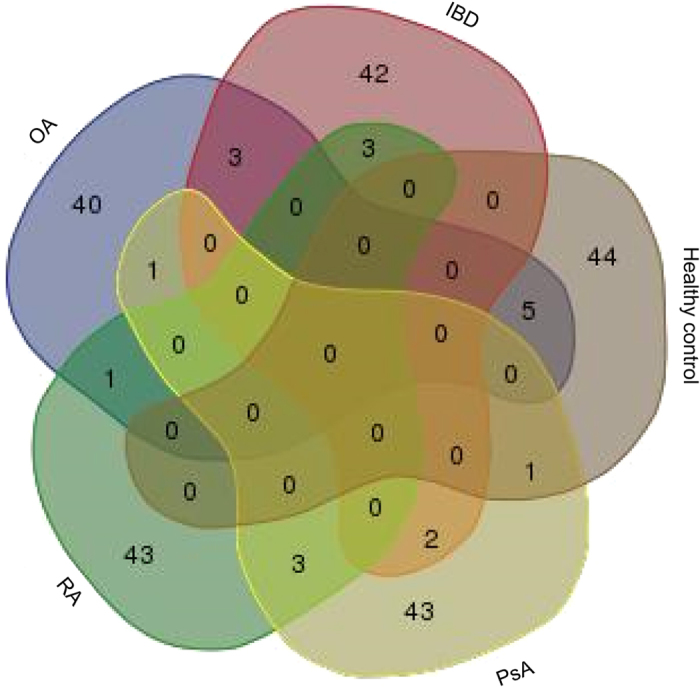
Venn diagram of the overlap between the 50 most significant peptides per group. Only minor overlap can be detected between the five groups, with 80% or more of the peptides being significant for one specific group only. OA – osteoarthritis; IBD – inflammatory bowel disease; PsA – psoriatic arthritis; RA – rheumatoid arthritis

Since potential peptide biomarkers specific for each of the 5 groups could be identified, we developed classifiers for each of the 5 groups. To avoid introducing bias, and to give similar “weight” to each group, we employed the same number of potential biomarkers (i.e. the 50 most significant peptides) as biomarkers in each group. This number was chosen based on of the number of potentially significant biomarkers available in all groups and the previous observation that more biomarkers confer higher stability to the classifier. There was very little overlap in the biomarkers between the five groups, with >80% of the biomarkers for each group being unique for that respective disease group only, as evident from the Venn diagram (Fig. 3). The 50 most significant biomarkers per group are listed in the Supplementary Table 2, including the average abundance of the peptide in each of the 5 groups separately, and the p-value. All 50 potential biomarkers were initially included in each of the high-dimensional classifiers, which were subsequently trained in the discovery cohort. Using a bootstrapping approach based on take-one-out cross-validation, the number of biomarkers employed was reduced to 45 in each classifier. This is based on past experience where a 10% reduction of the number of biomarkers was found to be the optimum between improving the classifier, and overfitting (Mischak, unpublished, and ref. 14).

**Figure 4:**
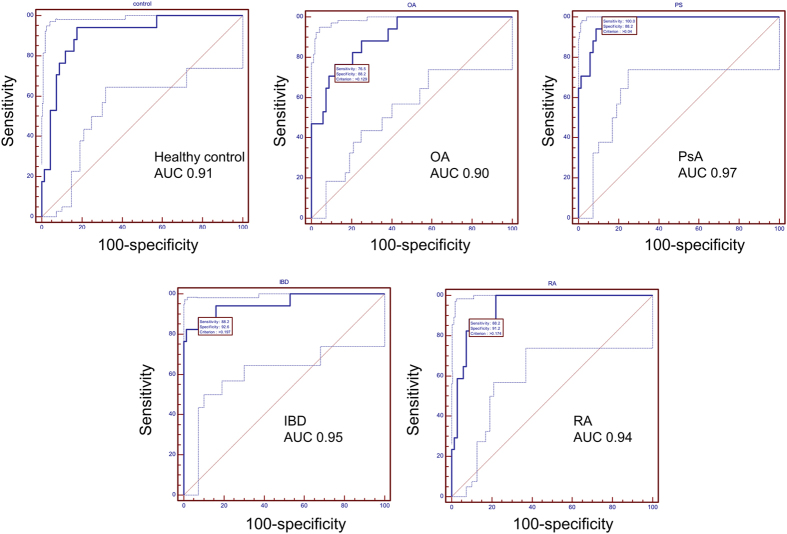
Receiver operating characteristics (ROC) curve for the performance of the five classifiers (as identified in the discovery (training) test set (n = 33 for each cohort) in the blinded independent validation test set (n = 75 in total, 17 for each cohort). The dotted lines represent the upper and lower limits of the 95% CI. AUC – area under the curve; OA – osteoarthritis; PsA – psoriatic arthritis; IBD – inflammatory bowel disease; RA – rheumatoid arthritis.

The five classifiers were subsequently applied to the validation set in a blinded manner. After unblinding, the results were evaluated using Receiver Operating Characteristics (ROC) analysis. As presented in Fig. 4, the classifiers enabled detection of the respective disease with very good accuracy, with the AUC being between 0.90 and 0.97, depending on the group. The scoring of each of the samples with all five classifiers is also presented in Supplementary Table 3. When examining the misclassified samples, no obvious association of the misclassification with a specific pathology could be observed, suggesting the errors are random.

To examine if the results can be linked to molecular pathophysiology, we investigated the biomarkers on a single peptide level. The ten most significant sequenced peptides for each of the five groups are presented in Table 2. This number was chosen for pragmatic reason, to allow for some coverage, while at the same time avoid investigating a very large list of peptides.

Collagen fragments are abundant in urine. 50% of the peptide markers associated with the different arthritic disease groups were collagen fragments, with collagen alpha-1(I) chain being the most frequently found species. The OA group displayed the highest number of reduced urinary collagen alpha-1(I) chain fragments (3/10), while in the healthy control group the highest number of increased collagen alpha-1(I) chain fragments (4/10) was observed. Two collagen alpha-1(I) chain fragments overlapped between these two conditions. All other collagen fragments, including collagen alpha-2(I), collagen alpha-1(II), collagen alpha-1(III), collagen alpha-5(IV), collagen alpha-1(VIII) and collagen alpha-2(IX) chains, did not display specific enrichment in any one of the groups. Only a few fragments were derived from proteins generally known to be associated to inflammation, including protein S100-A9, fibrinogen and ICAM5. In addition, a number of other peptide fragments derived from functionally very different proteins not previously identified to be associated to arthritis were identified (Table 2).

## Discussion

The identification of urinary biomarkers that distinguish between these discrete rheumatic and inflammatory diseases in this pilot study is encouraging, and suggests that further study of urinary peptidomics will be worthwhile to further explore the diagnostic potential of this approach for inflammatory arthritis. However, this study was small and will require validation in independent cohorts. If the utility of the classifiers is confirmed, it will also be necessary to establish whether changes in the sample collection protocols affect the accuracy of the various classifiers. Nevertheless, our study provides the first substantive data suggesting that inflammatory arthropathies could yield unique peptide profiles of this kind.

Ultimately, the goal would be the development of a diagnostic kit that could be used in routine clinical practice. This may benefit from a reduced number of biomarkers. To investigate this issue, we reduced the classifiers to 10 biomarkers per condition. In each case the AUC dropped, although not significantly, and classification based on 10 biomarkers was consistently of slightly lower accuracy than classification based on 45 biomarkers. This was expected based on our previous experience; similar approaches in chronic kidney disease^4^ or cardiovascular disease^7^ also demonstrated that a reduction of the number of biomarkers leads to a reduction in the diagnostic accuracy. Two issues may be responsible for this observation: 1) the complexity of these diseases cannot be displayed by a limited set of features, and 2) biological variability can to some degree be counteracted by assessment of multiple variables. Hence, a higher number of biomarkers will confer more stability and higher accuracy to the classifier.

It was possible that we would detect non-specific biomarkers of joint damage, or chronic inflammation rather than disease-specific biomarkers. We used a range of chronic disease with different but overlapping characteristics to mitigate this pitfall – for example, RA and PsA are both classical chronic inflammatory arthropathies, whereas OA compromises articular compromise but is not associated with significant systemic inflammation. We were able to develop classifiers that identified and distinguished patients with each of the selected diseases, indicating that the peptides included in these classifiers are not simply non-specific markers of either chronic inflammation or joint damage. This is also supported by the fact that few commonly known inflammatory markers (eg fibrinogen, protein S100-A9 and ICAM5) were among the 10 most significant biomarker peptides per disease group (Table 2). In contrast, peptide fragments of proteins involved in specific inflammatory processes not previously identified in arthritic diseases were found to be associated with specific disease groups. As an example, homeobox protein cut-like 1 (CUX1) was found to be associated to the RA group and has previously been shown to be potentially involved in pro-inflammatory tumour necrosis factor alpha (TNFα) production following lipopolysaccharide administration^15^. Collagen remodelling is key in articular disease^16^ and 50% of the peptides were fragments of various collagens. In addition, CUX1 has been shown to inhibit high dose transforming growth factor β-induced collagen I production^17^. Although the *in situ* change in abundance of the different urinary peptide markers remain to be determined, examination of the nature of the peptides might thus led to confirmation of existing processes in arthritic diseases or uncover novel insight. It should be noted that several of the biomarker peptides identified are from highly abundant proteins. The most plausible hypothesis for their differential abundance is a significant difference in specific proteases. Hence, the peptide biomarkers observed likely do not reflect abundance of the parental protein, but activity of potentially disease-specific proteases.

The results also suggest that this approach may have potential for the identification of patients with inflammatory arthritis in general when combining the disease classifiers (i.e. as screening test in primary care settings) as well as diagnostic test for the specific condition (i.e. in specialist secondary care settings). The latter may become increasingly important as the targeted biologic therapies for these conditions progressively diverge in response to increased understanding of the shared and distinct pathophysiologies between RA and PsA. While the patients with OA had end-stage disease requiring joint replacement, it would be interesting to evaluate the performance of the OA classifier in patients with early radiographic changes, particularly as the development of therapeutics in OA is severely hampered by the current absence of reliable diagnostic biomarkers.

It is not possible to directly compare the peptides identified in this study with those in previously published studies for a number of reasons. Firstly, the vast majority of previous studies evaluated peptides in serum, synovial fluid or tissue (for example cartilage, skin, synovial biopsies)^18^,^19^,^20^. Biomarkers present in these specimens would not be expected to be present in urine in an unmodified manner. These previous studies also focused largely on using proteomics to identify biomarkers of disease activity or treatment response rather than for diagnostic purposes^18^,^19^,^20^,^21^. Furthermore, the differentially expressed peptides identified will depend on which condition they are being compared to, and in our case this was a unique combination of four other conditions for each condition.

Our study has several limitations: firstly, not all biomarkers could be identified. This is most likely a result of unknown post-translational modifications^22^. However, a large number of biomarkers could be sequenced, and shows good linkage to molecular pathophysiology. Another limitation is the relatively small size cohort employed for validation. However, the classification results obtained are of very high statistical significance, suggesting a high certainty in the validity of the findings. Before considering employing these classifiers in routine clinical assessment of patients, their value has to be demonstrated and validated in a separate longitudinal study in a larger, carefully phenotyped cohort of patients with early or undifferentiated disease. These larger studies would also allow the influence of other factors (such as gender, age, body mass index (BMI), medication, disease activity) to be assessed, which is not possible in a study of the current size.

In summary, urine proteomics offers potential as a method for developing biomarkers in chronic rheumatic conditions. We have identified classifiers that can reliably differentiate between each of the five disease states. Furthermore, the differentially expressed peptides may suggest proteases differentially involved in, or affected by, the various conditions. While identifying the specific pathways is not required for the development of diagnostic biomarkers, further identification is likely to be useful for understanding the pathophysiology of these diseases and may have therapeutic implications. We plan to develop these aspects further in our future work.

## Methods and Materials

### Patients and sample collection

Patients with OA, PsA, RA or IBD attending hospital-based specialist outpatient clinics were invited to provide a sample for this study. The patients had a diagnosis made by their rheumatologist (RA and PsA), orthopaedic surgeon (OA) or gastroenterologist (IBD). Patients could be on any anti-rheumatic therapies excluding cyclosporine or biologic agents. Spot urine samples were collected, immediately filtered and chilled, and then stored frozen until processed as recommended by the European Kidney and Urine Proteomics, and the Human Kidney and Urine Proteome Project, and described previously^23^. All samples were analyzed with the investigator blinded to the patients’ diagnosis. The samples from the patients with RA were selected from patients in the Scottish Early RA (SERA) inception cohort biobank^24^ who fulfilled the 2010 ACR/EULAR diagnostic criteria for RA. Samples for OA patients were obtained from patients with OA, but no history of RA or PsA, attending orthopaedic pre-operative assessment clinics in advance of knee or hip replacement surgery. Samples for IBD were collected from patients with IBD but no joint disease. The SERA study (RA patients) was given study-specific approval by the West of Scotland Research Ethics Committee (REC reference number 10/S0704/20). The PsA, OA IBD and healthy control samples were collected using generic sample collection approval from the West of Scotland Research Ethics Committee (REC reference number 11/S0704/7). Our study was done according to the protocol approved by the research team in the Institute of Infection, Immunity and Inflammation, University of Glasgow, and in line with the Declaration of Helsinki and International Committee on Harmonisation good clinical practice. All patients provided written informed consent for their samples to be used for research.

### Urinary proteome analysis and peptide identification

The urine samples were prepared and analyzed using a P/ACE MDQ capillary electrophoresis system (Beckman Coulter, Fullerton, USA) on line coupled to a MicroTOF MS (Bruker, Bremen, Germany) exactly as described previously^10^. Details on accuracy, precision, selectivity, sensitivity, reproducibility, and stability of the CE-MS method have been previously described^10^,^25^. To ensure appropriate quality of the analytical platform, a standard urine sample^23^ is prepared and analyzed daily, and data are evaluated to fulfil the predefined quality control criteria, including appropriate reproducibility, as detailed previously (e.g. in Stalmach *et al*.^26^). MosaiquesVisu was used to analyse the CE-MS data^27^. Twenty nine collagen peptides that have been identified previously as being of high abundance and without apparent association to disease were used as internal standards^11^. Briefly, ion counts are employed as measure of relative abundance, and are calibrated based on the abundance of the 29 predefined internal standards, using linear regression analysis^11^. This approach has been tested extensively and used in over 100 studies and over 10000 samples^28^, and is being routinely employed in a number of large randomized controlled trials^29^,^30^,^31^. All detected peptides were deposited, matched, and annotated in a MicrosoftSQL database^26^, allowing for further analysis and comparison between case and control groups. Sequencing of target peptides was performed as described^32^, using Dionex Ultimate 3000 RSLS nano flow system (Dionex, Camberly UK) and a Beckman CE, coupled to an Orbitrap Velos MS instrument (Thermo Scientific).

### Statistical analysis and classifier development

After testing for normal distribution, continuous data were compared by Wilcoxon t-test, as this test has proven to be of superior statistical power in proteomics datasets^14^. A p-value of <0.05 was considered to be statistically significant. In order to control for the false discovery rate, the p-values were adjusted by the Benjamini and Hochberg method^33^. For the development of classifiers, the amplitudes of the single peptides that were selected as biomarkers were combined using a support vector machine (SVM), to result in a single variable (SVM score)^14^. The process of statistical analysis and subsequent classifier development has been previously optimized specifically for urinary peptide data from CE-MS analysis^14^ and has been applied as routine procedure in multiple studies, some also jointly with regulatory agencies^34^, and acknowledged as being of significant value by regulators^35^. We employed a study design based on an initial discovery cohort employed as training set for the classifier, and another test cohort to assess the performance of the biomarkers. Participants were randomly assigned, using an automated algorithm (the srand() function in C++), to either the training set (containing 2 thirds of the entire cohort; 33 samples from each of the four disease and the healthy control groups) or a validation test set (containing 1 third of the cohort; n = 17 per group).

## Additional Information

**How to cite this article**: Siebert, S. *et al*. Urinary proteomics can define distinct diagnostic inflammatory arthritis subgroups. *Sci. Rep.* **7**, 40473; doi: 10.1038/srep40473 (2017).

**Publisher's note:** Springer Nature remains neutral with regard to jurisdictional claims in published maps and institutional affiliations.

## Supplementary Material

Supplementary Table Legends

Supplementary Table 1

Supplementary Table 2

Supplementary Table 3

## Figures and Tables

**Table 1:** Clinical and demographic data for the disease groups.

Diagnosis (n = 50 for each group)	Age mean (±SD) years	Females number (%)
Healthy Controls	48.3 (±13.3)	33 (66%)
Inflammatory Bowel Disease (IBD): - Crohn’s (n = 42), ulcerative colitis (n = 8)	48.1 (±18.0)	28 (56%)
Osteoarthritis (OA)	64.6 (±10.3)	31 (62%)
Rheumatoid Arthritis (RA)	56.1 (±13.8)	33 (66%)
Psoriatic Arthritis (PsA)	53.8 (±11.7)	30 (60%)

**Table 2:** Most significant sequenced peptides associated with RA, PsA OA, IBD, or healthy controls.

OA		IBD		RA		PsA		Healthy control		Sequence	Protein Symbol	Protein Name
p-value	fold-change	p-value	fold-change	p-value	fold-change	p-value	fold-change	p-value	fold-change			
1.22E-06	0.12							1.16E-07	2.76	GRYVPGSASmGTTMAGVDPFTGNSAYRSAAS	PLAA	Phospholipase A-2-activating protein; PLAA
								3.97E-06	2.38	SGSVIDQSRVLNLGP	UMOD	Uromodulin
								1.23E-05	1.75	KEGGKGPRGETGPAGRpGEVGpPGPpGP	COL1A1	Collagen alpha-1(I) chain
8.79E-06	0.64							1.51E-05	1.38	TGSpGSpGPDGKTGPPGpAG	COL1A1	Collagen alpha-1(I) chain
4.40E-06	0.47							2.19E-05	1.75	DDGEAGKpG	COL1A1	Collagen alpha-1(I) chain
								2.84E-05	1.73	TTLSPSSSTTHEGEPTTFQSWPSSKDTSPAPSG	MUC12	Mucin-12
								3.55E-05	2.68	TGLSmDGGGSPKGDVDP	FXYD2	Sodium/potassium-transporting ATPase subunit gamma
								4.36E-05	2.07	ADGQPGAKGEpGDAGAKGDAGPpGPAGPAGPPGPIG	COL1A1	Collagen alpha-1(I) chain
								4.74E-05	1.79	TGEVGAVGppGFAGEKGPSGEAGTAGpPGTpGPQG	COL1A2	Collagen alpha-2(I) chain
								9.63E-05	2.48	QKGDEGPPGISIpGppGLDGQpGAP	COL4A5	Collagen alpha-5(IV) chain; COL4A5
						7.70E-06	1.59			AGSEADHEGTHSTKRG	FGA	Fibrinogen alpha chain; FGA
						9.72E-06	2.41			FVESQKDPENSPV	CTSA	Cathepsin A; CTSA
						2.30E-05	0.46			AGVANALAHKYH	HBD	Hemoglobin subunit delta
						2.88E-05	0.34			NVGApGAKGARGSAGPpGATGFpGAAGRVGPpGP	COL1A1	Collagen alpha-1(I) chain
						4.17E-05	7.55			LNAADADVPLDDLTFT	FREM2	FRAS1-related extracellular matrix protein 2; FREM2
						6.43E-05	0.22			AGGGAGGAAGAEGGPEAAGGAAESPAEGE	ICAM5	Intercellular adhesion molecule 5
						8.18E-05	0.22			KLGHPDTL	S100A9	Protein S100-A9
						8.37E-05	1.54			LGPHAGDVEGHLS	APOA4	Apolipoprotein A-IV
						9.58E-05	1.29			GppGPDGNKGEpG	COL1A2	Collagen alpha-2(I) chain
						1.18E-04	1.46			DKGETGEQGDRG	COL1A1	Collagen alpha-1(I) chain
				2.88E-09	5.37					PEPAKSAPAPKKG	HIST1H2BK	Histone H2B type 1-C/E/F/G/I
				1.97E-08	4.59					pGPQGPLGKPGAPGEPGPQG	COL8A1	Collagen alpha-1(VIII) chain
				8.62E-08	1.44					NGApGNDGAKGDAGApGApGSQGApG	COL1A1	Collagen alpha-1(I) chain
				1.63E-07	9.42					DGAKGDAGPAGPKGEpGSpGENGApG	COL1A1	Collagen alpha-1(I) chain
				4.53E-07	2.72					DGQPGAKGEpGDAG	COL1A1	Collagen alpha-1(I) chain
				7.21E-07	3.73					RVLNLGPITR	UMOD	Uromodulin; UMOD
				8.61E-07	2.28					PVQGQQQGP	CUX1	Homeobox protein cut-like 1; CUX1
				1.07E-06	2.04					NGEpGGKGERGApGEKGEGGppG	COL3A1	Collagen alpha-1(III) chain; COL3A1
				1.09E-06	3.46					DEAGSEADHEGTHSTK	FGA	Fibrinogen alpha chain
		9.99E-05	0.27	1.10E-06	2.47					PPPLPPPPPPPPP	PRIMA1	Isoform 2 of Proline-rich membrane anchor 1
		5.48E-08	0.25							GRAGEpGLQGpAGPPGEKGEpGDDGPSGAEGpP	COL2A1,	Collagen alpha-1(II) chain
		7.74E-08	0.55							EpGSpGENGApGQmGPR	COL1A1	Collagen alpha-1(I) chain
		4.79E-07	0.33							GADGQPGAKGEpGDAGAKGDAGPpGPAGP	COL1A1	Collagen alpha-1(I) chain
		2.50E-06	0.41							GEVGPAGSpGSNGApGQRGEpGPQGHAGAQGp	COL3A1	Collagen alpha-1(III) chain; COL3A1
		8.44E-06	0.43							PpGppGPpGVPGSDGIDGDNGPPGK	COL9A2	Collagen alpha-2(IX) chain; COL9A2
		1.12E-05	2.13							GQDGRpGPpGPpG	COL1A1	Collagen alpha-1(I) chain
		1.61E-05	0.26							PAPAPPPEPERPKEVE	MYL3	Myosin light chain 3; MYL3
		1.98E-05	0.16							SIAAGGEGLTDVSPE	ATG12	Ubiquitin-like protein ATG12; ATG12
		2.29E-05	2.83							NTGAPGSpGVSGpKGDAGQPGEKGSpGAQGPPGAPGPLG	COL3A1	Collagen alpha-1(III) chain
		3.56E-05	0.50							SVIDQSRVLNLGPITR	UMOD	Uromodulin
1.33E-07	2.29									ASTAQASSSAASNNHQVGSGNDPWSA	SNX9	Sorting nexin-9
1.79E-06	1.39									FAERNPVEELTVDSPPVQ	PCDH12	Protocadherin-12
1.69E-05	1.45									PpGEAGKpGEQGVpGDLGAPGP	COL1A1	Collagen alpha-1(I) chain
2.26E-05	0.62									LTGPIGPPGpAGApGDKGESGPSGPAGPTG	COL1A1	Collagen alpha-1(I) chain
3.78E-05	1.72									HQGPAGPpGPpGPpGPpGVSGGG	COL1A2	Collagen alpha-2(I) chain
3.84E-05	1.55									VANEESEHNQGASEENGLP	TRPC4AP	Short transient receptor potential channel 4-associated protein
3.84E-05	1.90									SGPpGPDGNKGEpG	COL1A2	Collagen alpha-2(I) chain
