# Supplementary material for: Urinary proteomics can define distinct diagnostic inflammatory arthritis subgroups
Source: Sci Rep. 2017 Jan 16;7:40473. doi: 10.1038/srep40473 (PMC5320079; doi:10.1038/srep40473)
Supplement: Supplementary Table Legends [file srep40473-s1.pdf]

Urinary proteomics can define distinct diagnostic inflammatory arthritis subgroups

Stefan Siebert*, Duncan Porter, Caron Paterson, Rosie Hampson, Daniel Gaya, Agnieszka Latosinska, Harald Mischak, Joost Schanstra, William Mullen , Iain McInnes

SUPPLEMENTARY TABLE LEGENDS

Supplementary table 1: Peptides significantly associated with the each of the five groups. Each disease group was tested against the combined other four groups. All peptides that reached significance are listed with mass, migration time, unadjusted p-value, p-value adjusted according to Benjamini and Hochberg (BH), as well as sequence and parental protein, if available.

Supplementary table 2: The 50 most significant biomarkers in each group are listed, indicating distribution of each of these biomarkers in the 5 groups. Where known, the peptide sequence is given. P-values are listed only on the cases where the peptides were found to be among the 50 most significant group-specific peptides. Upregulation is indicated by a red label, reduced abundance by a blue label. Evidently, very little overlap between the groups can be observed.

Supplementary table 3: Scoring of all samples in the validation test set with all 5 classifiers developed. Samples with incorrect assignment are labelled yellow.
